# Gene expression regulated by abatacept associated with methotrexate and correlation with disease activity in rheumatoid arthritis

**DOI:** 10.1371/journal.pone.0237143

**Published:** 2020-08-06

**Authors:** Céline Derambure, Gaelle Dzangue-Tchoupou, Maria Antonietta D’Agostino, Thierry Lequerré, Olivier Vittecoq

**Affiliations:** 1 Normandie Univ, UNIROUEN, Inserm U 1245, Rouen, France; 2 Normandie Univ, UNIROUEN, Inserm U 1234, Rouen, France; 3 Department of Rheumatology, AP-HP Ambroise Paré Hospital, University of Versailles Saint Quentin en Yvelines, Boulogne-Billancourt, France; 4 Department of Rheumatology Inserm CIC/CRB1404, Rouen University Hospital, Rouen Cedex, France; Nippon Medical School, JAPAN

## Abstract

**Objectives:**

Abatacept acts as a competitive inhibitor of the CD28/(CD80/86) costimulation signal required for T cell activation. Mechanisms of action of abatacept have not been fully investigated. The objective of this study was to provide detailed insight into the mode of action of Abatacept based on gene expression data.

**Methods:**

In this ancillary study from the APPRAISE trial, we investigated the global molecular effects of Abatacept in whole blood samples collected prospectively in biologic naive rheumatoid arthritis patients (n = 19) at baseline and 6 months after the initiation of Abatacept therapy concomitant with methotrexate. Whole human genome microarrays (4x44K) were performed on both baseline and 6-month samples from responders and non-responders patients categorized according to EULAR criteria. T-test with Benjamini-Hochberg correction was performed to identify significant gene expression changes. Gene Ontology and Single Experiment Analysis tools allowed us to highlight specific biological mechanisms involved in methotrexate/Abatacept.

**Results:**

In methotrexate/Abatacept responders, 672 genes were significantly (q<0.05) dysregulated at 6 months compared to baseline. Correlation analysis highlighted 19 genes whose dysregulations were significantly associated with disease activity variation (p<0.05) and whose functions were associated with proliferation, apoptosis of cells and mitochondrial metabolism, suggesting a restoration of oxidative signaling. The other 653 gene expression changes were relative to direct or indirect effects of methotrexate/Abatacept treatment and were significantly (p<0.005) involved in pathways relative to mRNA processing, proteasome, angiogenesis, apoptosis and TCR signaling. This study highlights new mechanisms of action of methotrexate/Abatacept and may provide new therapeutic targets to prevent autoimmunity in rheumatoid arthritis.

## 1. Introduction

Abatacept (ABA) is indicated for the treatment of adult patients with moderately to severely active RA. ABA may be used as monotherapy or concomitantly with disease-modifying antirheumatic drugs (DMARDs) such as methotrexate (MTX). ABA is a soluble human recombinant fusion protein for which the extracellular domain of human cytotoxic T lymphocyte-associated molecule 4 (CTLA-4Ig) is bound to the Fc portion of human IgG1. ABA decreases the T cell responses involved in RA pathophysiology. In fact, T cells are activated by the engagement of the T cell receptor (TCR) and the interaction of costimulatory molecules, such as CD80/CD86 on antigen presenting cells (APC), with CD28 on T cells. Activated T cells express T-lymphocyte antigen-4 (CTLA-4), which binds both costimulatory molecules CD80 and CD86, but in contrast to CD28 binding, delivers anti-proliferative signals that downregulate T cell activation [1–3]. Thus, CD80 and CD86 appear to present dual functions within the immune system: activation signals by their interaction with CD28 as well as inhibition signals by their interaction with CTLA-4. Therefore, we sought to understand how ABA can act on both APC and T cells when ABA interacts with CD80/CD86.

Overall, CTLA-4Ig limits T cell proliferation, promotes T cell tolerance, induces reverse signaling in antigen-presenting cells, reduces the migratory capacity of monocytes decreasing the expression of several adhesion molecules, induces the 2,3-dioxygenase indolamine pathway in dendritic cells and heme oxygenase in Tregs, restores T cell small GTPase Ras-related protein 1 (Rap1) function and inhibits osteoclast precursor cell differentiation [4–7]. Despite some data available on synovium, the mechanisms of action of ABA have not been fully investigated at the molecular level [8, 9]. Gene expression signatures can provide an unbiased view of the molecular changes underlying biologically and medically interesting phenotypes.

In this ancillary study from the APPRAISE trial, we investigated the global molecular effects of ABA therapy concomitant with MTX in whole blood samples collected prospectively in biologic naive RA patients at baseline and 6 months after the initiation of therapy. The objective of this study was to provide detailed insight into the mode of action of ABA based on gene expression data on whole blood.

## 2. Materials and methods

### Patients from the APPRAISE trial

A total of 19 RA patients from the APPRAISE trial (NCT00767325) were enrolled in this ancillary study [10, 11]. Eligible patients were ≥18 years of age, had American College of Rheumatology (ACR)-defined RA for at least 6 months according to the 1987 classification criteria [12], and were receiving MTX (≥15 mg/week) for at least 3 months prior to baseline. Patients were required to have active disease, defined by a baseline Disease Activity Score 28 (DAS28-CRP) of >3.2 or tender and swollen joint counts of ≥6 and a C reactive protein (CRP) level greater than the normal upper limit. All patients received intravenous infusions of ABA (10 mg/kg) at baseline, and at weeks 2, 4, 8, 12, 16, 20 and 24, in addition to stable doses of concomitant MTX (≥15 mg/week). Oral corticosteroid use (stable dose of ≤10 mg prednisone/day) was permitted during the study. For this study, 5 ml of whole blood were collected in PAXgene RNA tube (Qiagen, PreAnalytiX, GmbH, Courtaboeuf, France) just before the first infusion and 6 months later and stored at -80°C until use.

The study was approved by the Institutional Review Board/Independent Ethics Committee (IRB/IEC) and local ethics committees (Hopital Ambroise Pare, Comité de Protections des Personnes Idf Vlll Lab D’Anatomopathologie, 9 Ave Charles De Gaulle, Boulogne-Billancourt 92100, France. Comitato Etico Per La Sperimentazione Del Farmaco, Asur, Territoriale 5 Di Jesi Via Gallodoro 68, Jesi (An) 60035, Italy. Azienda Ospedaliera Universitaria Policlinico G. Martino, Comitato Etico Scientifico Via Consolare Valeria, 1, Messina 98124, Italy. Leeds East Research Ethics Committee, Yorkshire and Humber Rec Cntr Off. First Floor, Millside Mill Pond Lane, Leeds, England LS6 4RA, United Kingdom. Ceic Fundacio Unio Catalana D’Hospitals, Area De Serveis C/Bruc 72–74 1a, Barcelona 08009, Spain. Azienda Universitaria Senese, Comitato Etico Locale La Sperimentazione Clinica Dei Medicinali Farmacia Aous—Viale Bracci, Siena 53100, Italy. De Videnskabsetiske Komiteer For Region Hovedstaden, Kongens Vaenge 2, Hillerod 3400, Denmark. Com Sperimentazione Clin Dei Med Azienda Osp-Univ Pisana, Via Roma 67, Pisa 56126, Italy. Comitato Etico Dell’Universita Cattolica Del Sacro Cuore, Policlinico Universitario Agostino Gemelli Di Roma Largo A Gemelli 8, Roma 00168, Italy. Rek Sorost, Frederik Holsts Hus Ulleval Terrasse Ulleval Sykehus Kirkeveien 166, Oslo 0450, Norway. Ceic—Fundacion Jimenez Diaz-Ute, Avda. Reyes Catolicos, 2-2a, Madrid 28040, Spain. Hospital Mostoles, Ceic Area 8 C/Rio Jucar S/N, Madrid 28935, Spain. Universita Di Roma La Sapienza, Comitato Etico Dell’Azienda Policlinico Umberto I, Roma 00161, Italy. Ceic Area 9-Hosp Severo Ochoa De Leganes Y Hosp De Fuenlabrad, Avenida Orellana S/N Leganes, Madrid 28911, Spain. Hospital Universitario La Princesa, C/Diego De Leon, 62, Madrid 28006, Spain. Farmakologiai Etk Bizottsaga, Arany J.U. 6–8., Budapest 1051, Hungary. Comitato Etico Per La Sperimentazione Dell’Azienda, Ospedaliera Istituti Ospitalieri Di Verona Piazzale Stefani 1, Verona 37126, Italy. Ethikkommission Der Ludwig-Maximilians Universitaet, Marchioninistr. 15, Muenchen 81377, Germany) and was conducted in accordance with the ethical principles underlying the European Union Directive 2001/20/EC and the United States Code of Federal Regulations on Good Clinical Practice as defined by the International Conference on Harmonisation. All patients provided written informed consent.

### Clinical evaluation and response to MTX/ABA

Clinical characteristics were collected at baseline and 6 months later: age, gender, disease duration, MTX and corticosteroid doses. All the patients were treated with MTX concomitant with ABA whereas 52.6% of the patients (10/19) received corticosteroids. Anti-citrulinated peptide antibody (ACPA) and rheumatoid factors titers were not collected in this study. The response to MTX/ABA was evaluated at 6 months with EULAR response criteria based on DAS28-CRP [13]. These 19 RA patients were selected among the patients from the APPRAISE trial on the basis of their highest amplitude of response for good responders (change in DAS28-CRP ≥2.3) after 6 months of treatment (n = 14). Only five non responders were at our disposal (S1 Fig).

### RNA preparation

Total RNAs from whole blood were extracted with PAXgene blood RNA kit according to the manufacturer’s recommendations (Qiagen PreAnalytiX GmbH, Courtaboeuf, France) and stored at -80°C until use. The quality and quantity of isolated RNAs were assessed using the 2100 Bioanalyzer (Agilent Technologies, Santa Clara, *California*, USA) and the Nanodrop (Thermo Scientific, *Wilmington*, USA).

### Microarrays

Whole human genomic DNA microarrays were used to analyze two-colored gene expression profiling (4x44K Whole Human Genome, Agilent Technologies, Les Ulis, France). To identify dysregulated gene expression under treatment, baseline samples (100ng) were Cyanine-3 labeled and co-hybridized with their cyanine-5 paired samples after treatment (6 months) according to the manufacturer’s instructions (Low Input QuickAmp Labeling Kit, Agilent Technologies, Les Ulis, France). The microarrays were scanned with a 5μM pixel size using the DNA Microarray Scanner GB (Agilent Technologies, Les Ulis, France). Image analysis and extraction of raw and normalized signal intensities by Lowess method were performed with Feature Extraction Software 10.5.1.1 (Agilent Technologies, Les Ulis, France). The data were in agreement with the Minimum Information About a Microarray Experiment guidelines and were deposited in the National Center for Biotechnology Information’s Gene Expression Omnibus (GEO) database (https://www.ncbi.nlm.nih.gov/geo/). The data are accessible using the accession number GSE91079 for gene expression changes between baseline and 6 months under MTX/ABA. Data from a previous study were used to recover the ratio of gene expression from RA patients at baseline just before treatment compared to 10 healthy donors (GSE68215) [14].

### Statistical and functional analysis

Comparisons of clinical and biological data using GraphPad Prism software version 5.0 were performed using unpaired or paired Student’s t-test; unpaired t-test for age, methotrexate and corticosteroid doses, CRP, DAS28, TJC, SJC and VAS between R and NR at onset (0 month); paired t-test for differences between 0 and 6 months in both R and NR subsets; and Fisher’s exact test for frequency comparisons (sex-ratio).

Data from the transcriptomic analysis were analyzed with GeneSpring GX V.14.0 (Agilent Technologies, Les Ulis, France). A first step of filtration was applied to remove the spike-in probes, the flagged probes (not uniform, saturated or outlier) and the probes whose intensities were close to the background (<100 fluorescence intensity) both at baseline and at 6 months in R and NR separetely. Student’s t-test with Benjamini Hochberg correction to check the False Discovery Rate ((FDR), FDR = 0.05) was used to determine the statistical significance (q-value < 0.05) in gene expression levels between 6-month samples and paired-baseline samples. The Gene Ontology (GO) analysis was used to investigate the biological processes, molecular function or cellular localization enriched in the transcript list showing significant changes between 6 month and baseline. *P* value was computed by standard hypergeometric distribution. The GeneSpring Single Experiment Analysis (SEA) bio-informatics tool was used for computational analysis to identify potential curated canonical pathways which are enriched in the transcript list showing a significant gene expression difference between 6 month and baseline, using WikiPathways database (http://www.wikipathways.org). 760 pathways were evaluated and gene enrichment was measured by Fisher’s exact test.

For genes with a statistically significant change in expression between 6 month and baseline, correlation between change in gene expression and change in DAS28 was computed. Nominal significance of the Pearson’s correlation test was used to determine if the change in gene expression was related to ABA/MTX response.

## 3. Results

### Characteristics of RA patients and their response to MTX/ABA

From the APPRAISE trial, 19 RA patients were selected among R (n = 14) and NR (n = 5) treated by MTX/ABA according to EULAR response criteria. Table 1 provides demographic and clinical data for these 19 RA patients at baseline and after 6 months of treatment. The characteristics of RA patients from R and NR at baseline were comparable for all parameters suggesting absence of bias in patient selection (*p*>0.05). In the R subset, tender joint count, swollen joint count, global assessment of disease measured by the patient with visual analog scale, disease activity score (DAS28-CRP) (*p*<0.001) and CRP (*p*<0.05) improved significantly after 6 months of treatment, while in the NR subset no parameters improved significantly (Table 1).

**Table 1:** Clinical and biological parameters of RA patients.

	R (n = 14)		NR (n = 5)	
	0	6 months	0	6 months
Age	58.79 +/- 4.87		58.60 +/- 4.2	
Gender (F/M)	11/3		3/2	
Duration of disease (years)	4.5 +/- 1.75		8.80 +/- 4.13	
Methotrexate dose (mg/week)	15.36 +/- 1.11		18 +/- 1.23	
Corticosteroid dose (mg/day)	6 +/- 0.75		6.5 +/- 1.5	
Tender joint count/28	15.07 +/- 1.46 ***	0.43 +/- 0.17	9.60 +/- 4.35	10.80 +/- 2.96
Swollen joint count /28	12.78 +/- 1.66 ***	1.5 +/- 0.66	9.40 +/- 1.78	8.40 +/- 1.83
Patient assessment of disease (VAS scale/100 mm)	62.07 +/- 6.81 ***	17.14 +/- 4.9	49.4 +/- 12.29	49.0 +/- 12.77
DAS28-CRP	5.77 +/- 0.23 ***	2.09 +/- 0.12	4.95 +/- 0.76	4.97 +/- 0.68
CRP (mg/L)	13.83 +/- 4.83 *	2.91 +/- 0.53	20.47 +/- 8.50	16.55 +/- 9.03

Mean +/- Standard error of the mean. *P* values were determined by Student’s t-test or Fisher exact test (*: p<0.05; ***: p<0.0001). CRP: C reactive protein; DAS28: disease activity score 28; NR: non-responders; R: responders; VAS: visual analog scale. Data available in S3 Fig.

### Gene expression changes under MTX/ABA in R

**Fig 1:**
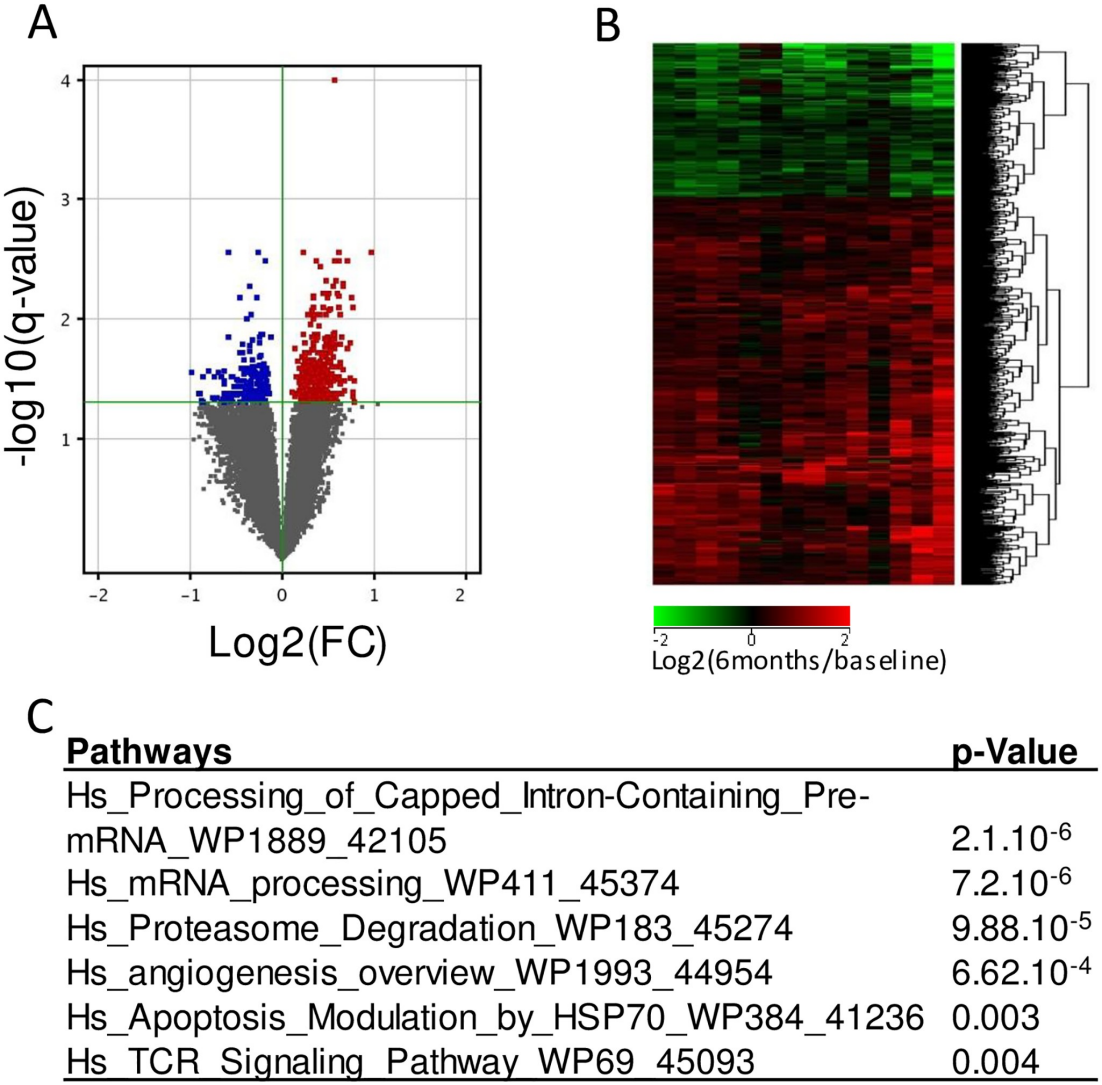
Gene expression changes between baseline and 6 months after MTX/ABA in responders. Baseline samples were co-hybridized with 6 months samples on 4x44K Agilent whole human genome microarrays. Data are expressed by a log_2_ ratio (6 months / baseline). **A**: Volcano plots in responders (n = 14) was built with a *t-test* with Benjamini-Hochberg correction for false discovery rate estimation. 672 genes were significantly dysregulated in R (q<0.05). Fold change (FC) is the ratio of relative abundance of transcripts in RA patients after treatment (6 months) *vs* before treatment (baseline). **B**: Hierarchical clustering (Pearson’s correlation coefficient and complete linkage metrics) showing the 481 up-regulated genes and the 191 down-regulated genes in responders between baseline and 6 months. **C**: single experiment analysis of the 653 genes whose dysregulations between baseline and 6 months were associated with MTX/ABA in responders. We identified 6 signaling pathways involved in MTX/ABA response from the WikiPathways database (enrichment p-value < 0.005).

**Fig 2:**
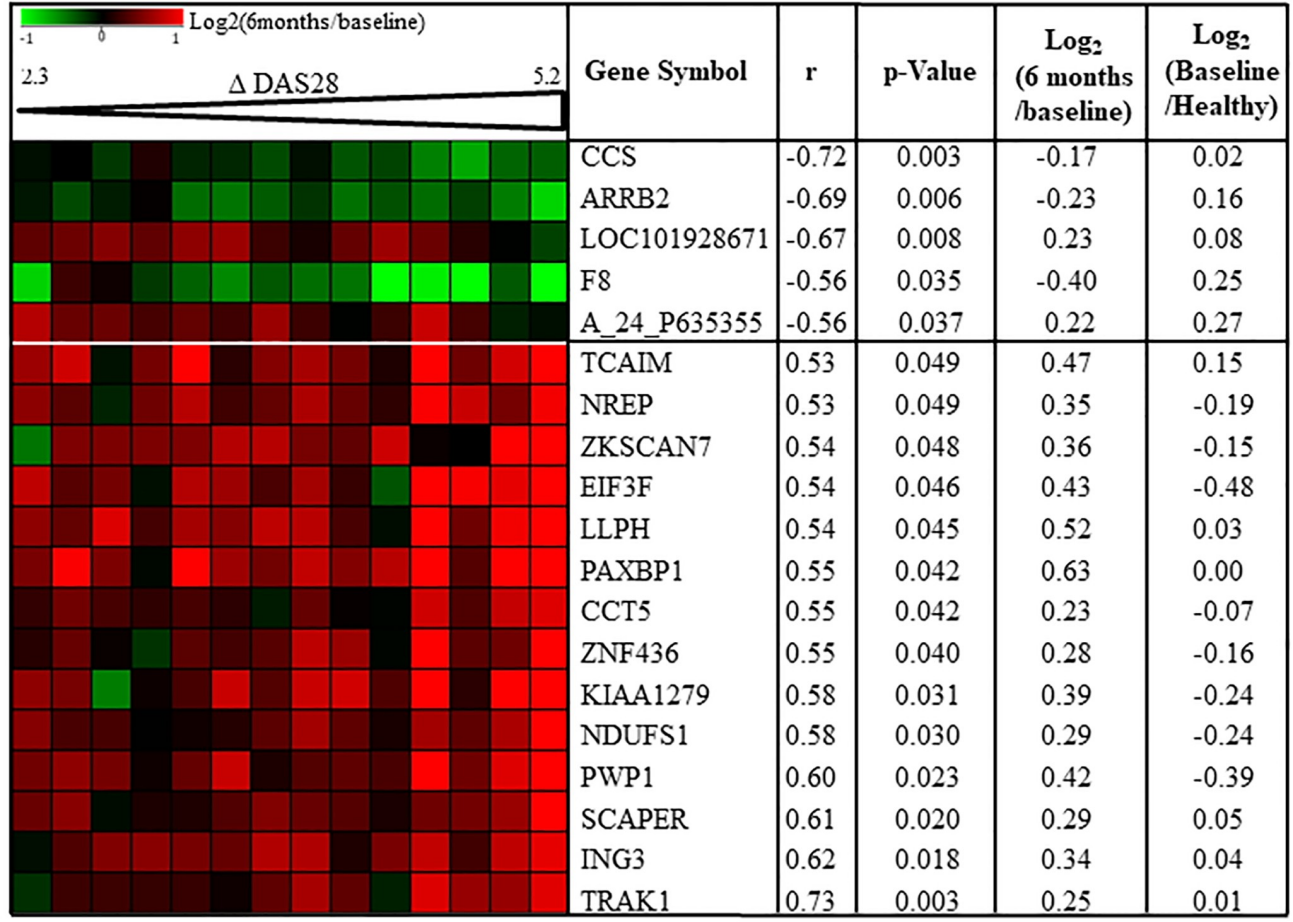
Disease activity correlates with gene expression changes between baseline and 6 months in MTX/ABA responders. Pearson’s correlation coefficients (r) were calculated for all 672 transcripts which were significantly dysregulated between baseline and 6 months after treatment in RA responders (n = 14). Nineteen transcripts were correlated (p<0.05) with change in disease activity score 28 (Δ DAS). For each transcript mean of Log_2_ (6 months / baseline) and mean of log_2_ (baseline / healthy controls) are indicated. A_24_P635355: Agilent probe ID; ARRB2: arrestin beta-2; CCS: copper chaperone for superoxide dismutase; CCT5: Chaperonin containing TCP1 subunit 5; EIF3F: eukaryotic translation initiation factor 3 subunit; F8: Coagulation factor VIII; ING3: inhibitor of growth family member 3; KIAA1279: KIF1 binding protein; LLPH: LLP homolog. long-term synaptic facilitation; NDUFS1: NADH-ubiquinone oxidoreductase 75 kDa subunit; NREP: neuronal regeneration related protein; PAXBP1: PAX3 and PAX7 binding protein 1; PWP1: Periodic tryptophan protein 1 homolog; SCAPER: S-phase cyclin A-associated protein in the endoplasmatic reticulum; TCAIM: T-cell activation inhibitor; TRAK1: Trafficking protein Kinesin binding 1; ZKSCAN7: Zinc finger with KRAB and SCAN domains 7; ZNF436: zinc finger protein 436.

After filtration steps, 21,529 transcripts in R were retained for subsequent differential gene expression analyses between baseline and 6 months. By t-test adjusted with Benjamini-Hochberg correction (5% FDR with *q*< 0.05) for multiple testing, 672 genes were significantly dysregulated in R between baseline and 6 months of treatment (Fig 1A). The size of NR subset of patients (n = 5) was too low and the analysis statistically underpowered to identify significant gene expression changes in this subset. Among the dysregulated genes in R, 481 genes were up-regulated while 191 genes were down-regulated under treatment (Fig 1B). In order to differentiate gene expression changes (log_2_(6-month expression/ baseline expression)) relative to both disease activity extent of improvement (Δ DAS28-CRP = DAS28-CRP at baseline—DAS28-CRP at 6 months) and/or treatment effects in R, correlation analysis was performed between gene expression changes and Δ DAS28-CRP between baseline and 6 months. Among the 672 genes dysregulated in R, 19 transcripts were correlated with DAS28 extent of improvement (*p* <0.05) (Fig 2, S2 Fig). The 653 other transcripts were not linked to disease activity extent of improvement suggesting maybe a gene expression change relative to direct or indirect treatment effects.

### Gene expression change relative to disease activity variation

Among the 19 genes dysregulated relative to disease activity between baseline and 6 months in R, 5 were negatively (-0.72 < r < -0.56) correlated with change in disease activity (increase in gene expression was correlated with worse response) and 14 were positively (0.53 < r < 0.73) correlated with change in disease activity (increase in gene expression was correlated with better response) (Fig 2, S2 Fig). Based on the findings of our previous study, half of these 14 genes, were down-regulated at baseline in RA responders to MTX/ABA compared to 10 healthy subjects (Fig 2). Conversely, for the expression of the 5 genes negatively correlated with disease activity change between baseline and 6 months, gene expression ratios between baseline in RA responders to MTX/ABA and 10 healthy subjects were all positive (Fig 2) suggesting that these 5 genes were up-regulated at baseline for RA responders compared to healthy subjects. The up or down regulation of these 19 genes compared to regulations between healthy subjects and responders suggest a trend toward the normalization of these gene expressions at 6 months compared to baseline. The analysis of these 19 transcripts with GO tool showed no biological process or molecular function enriched in this transcript set. Moreover, when these 19 mRNA were submitted to WikiPathways via SEA tool from GeneSpring software, we found a significant enrichment of 7 signaling pathways (p<0.05) but including just one gene out of 19 for each pathway. Therefore, these 19 transcripts cannot be mapped to specific biological function. These results allowed us to consider that the gene expression of these 14 genes increased and that of the 5 genes decreased to trend toward a normal gene expression level, when disease activity variation improved. These results are specific to R patients since the gene expression levels of these 19 genes did not change significantly in 5 NR between baseline and 6 months.

### Gene expression changes relative to ABA in responders

Among the 672 genes whose gene expression differed under MTX/ABA in R between baseline and 6 months, 653 were relative to ABA since their gene expression levels were dysregulated with no correlation with disease activity improvement (DAS28-CRP). These 653 transcripts were submitted to GO analysis showing a significant enrichment of 20 GO classes relative to nuclear localization and mRNA processes.

The WikiPathways analysis by SEA tool of these 653 genes led us to identify a significant enrichment of 6 signaling pathways involved in ABA response: 2 mRNA processes, proteasome, angiogenesis, apoptosis and TCR signaling (Fig 1C) (p<0.005). mRNA processing pathways were in accordance with GO analysis results. All these genes dysregulated under ABA and involved in these 6 pathways are indicated in Table 2.

**Table 2:** Transcripts relative to significantly enriched pathways in MTX/ABA responders.

ProbeName	Log_2_(FC)	Gene Symbol	Gene Name	RefSeqAcc	UniGeneID
**Hs_mRNA_processing_WP411_45374 (p = 2,1.10**^**−6**^**)**					
A_23_P61551	-0.26	CD2BP2	CD2 (cytoplasmic tail) binding protein 2	NM_006110	Hs.202677
A_23_P28688	0.28	CPSF3	cleavage and polyadenylation specific factor 3. 73kDa	NM_016207	Hs.515972
A_23_P37137	0.36	DNAJC8	DnaJ (Hsp40) homolog. subfamily C. member 8	NM_014280	Hs.433540
A_24_P517901	0.34	HNRNPA1	heterogeneous nuclear ribonucleoprotein A1	NM_002136	Hs.546261
A_32_P142028	0.25	HNRNPC	heterogeneous nuclear ribonucleoprotein C (C1/C2)	NM_031314	Hs.508848
A_24_P724984	0.62	HNRNPR	heterogeneous nuclear ribonucleoprotein R	NM_001102398	Hs.373763
A_24_P157424	0.52	NCBP2	nuclear cap binding protein subunit 2. 20kDa	NM_007362	Hs.591671
A_24_P151727	0.23	NONO	non-POU domain containing. octamer-binding	NM_007363	Hs.533282
A_23_P129614	0.24	NUDT21	nudix (nucleoside diphosphate linked moiety X)-type motif 21	NM_007006	Hs.528834
A_32_P193646	0.27	RBMX	RNA binding motif protein. X-linked	NM_002139	Hs.380118
A_23_P162945	0.25	SRP54	signal recognition particle 54kDa	NM_003136	Hs.167535
**Hs_Processing_of_Capped_Intron-Containing_Pre-mRNA_WP1889_42105 (p = 7.2.10**^**−6**^**)**					
A_23_P61551	-0.26	CD2BP2	CD2 binding protein 2	NM_006110	Hs.202677
A_23_P37137	0.36	DNAJC8	DnaJ (Hsp40) homolog. subfamily C. member 8	NM_014280	Hs.433540
A_24_P517901	0.34	HNRNPA1	heterogeneous nuclear ribonucleoprotein A1	NM_002136	Hs.546261
A_32_P77502	0.38	HNRNPA3	heterogeneous nuclear ribonucleoprotein A3	NM_194247	Hs.516539
A_32_P142028	0.25	HNRNPC	heterogeneous nuclear ribonucleoprotein C (C1/C2)	NM_031314	Hs.508848
A_24_P724984	0.62	HNRNPR	heterogeneous nuclear ribonucleoprotein R	NM_001102398	Hs.373763
A_24_P157424	0.52	NCBP2	nuclear cap binding protein subunit 2. 20kDa	NM_007362	Hs.591671
A_32_P193646	0.27	RBMX	RNA binding motif protein. X-linked	NM_002139	Hs.380118
**Hs_Proteasome_Degradation_WP183_45274 (p = 9.88.10**^**−5**^**)**					
A_24_P113674	0.49	HLA-B	major histocompatibility complex. class I. B	NM_005514	Hs.77961
A_23_P113716	0.49	HLA-C	major histocompatibility complex. class I. C	ND	Hs.743218
A_24_P311926	0.48	HLA-G	major histocompatibility complex. class I. G	NM_002127	Hs.512152
A_24_P418044	0.35	HLA-J	major histocompatibility complex. class I. J (pseudogene)	NR_024240	Hs.720762
A_32_P57870	0.20	PSMC1	proteasome (prosome. macropain) 26S subunit. ATPase. 1	NM_002802	Hs.356654
A_23_P109928	0.24	PSMD6	proteasome (prosome. macropain) 26S subunit. non-ATPase. 6	NM_014814	Hs.152536
A_24_P681301	0.26	UBC	ubiquitin C	NM_021009	Hs.520348
**Hs_TCR_Signaling_Pathway_WP69_45093 (p = 0.004)**					
A_24_P128145	0.32	ATF2	activating transcription factor 2	NM_001880	Hs.592510
A_24_P945262	0.35	CARD11	caspase recruitment domain family. member 11	ND	Hs.665701
A_23_P34676	0.36	CD247	CD247 molecule	NM_198053	Hs.156445
A_24_P407717	-0.17	GRB2	growth factor receptor-bound protein 2	NM_002086	Hs.444356
A_23_P159920	-0.24	IKBKG	inhibitor of kappa light polypeptide gene enhancer in B-cells	NM_003639	Hs.43505
A_23_P44112	0.24	LAT	linker for activation of T cells	NM_014387	Hs.632179
**Hs_Apoptosis_Modulation_by_HSP70_WP384_41236 (p = 0.003)**					
A_23_P72537	0.36	AIFM1/PDCD8	apoptosis-inducing factor. mitochondrion-associated. 1	NM_004208	Hs.424932
A_24_P29665	0.34	CYCS	cytochrome c. somatic	NM_018947	Hs.437060
A_23_P86917	-0.20	FADD	Fas (TNFRSF6)-associated via death domain	NM_003824	Hs.86131
**Hs_angiogenesis_overview_WP1993_44954 (p = 6.62.10–4)**					
A_24_P128145	0.32	ATF2	activating transcription factor 2	NM_001880	Hs.592510
A_24_P407717	-0.17	GRB2	growth factor receptor-bound protein 2	NM_002086	Hs.444356
A_23_P40174	-0.66	MMP9	matrix metallopeptidase 9	NM_004994	Hs.297413
A_23_P13969	0.41	PXN	paxillin	ND	Hs.446336
A_23_P107401	-0.32	TIMP2	TIMP metallopeptidase inhibitor 2	NM_003255	Hs.633514
A_23_P132611	0.56	VHL	von Hippel-Lindau tumor suppressor. E3 ubiquitin protein ligase	NM_000551	Hs.517792

FC: fold change (6months/baseline). RefSeqAcc: accession number in RefSeq database. Probe name: Agilent probe ID. ND: not determined.

## 4. Discussion

The main objective of this study was to investigate the molecular effects of ABA on gene expression in whole blood from RA patients. We compared the molecular changes under ABA by means of transcriptomic analysis of whole blood. From the APPRAISE trial, we selected 14 R and 5 NR to the MTX/ABA concomitant treatment. At baseline, all the 19 patients were comparable and had high disease activity (Table 1). After 6 months of treatments, DAS28 decreased from 5.8 ± 0.2 to 2.1 ± 0.1 in R, while it was unmodified in NR (from 4.9 ± 0.8 to 5 ± 0.7). We did not consider corticosteroids as a potential bias since both the proportion of patients treated in concomitance with corticosteroids and corticosteroids doses are not significatively different between R and NR subsets (p = 0.63 and 0.77 respectively).

In R, ABA significantly changed the gene expression level of 672 genes including 481 up-regulated and 191 down-regulated genes. The question arises as to whether the changes of the genes expressions results from the decrease of disease activity or from the direct or indirect effects of ABA. Only 19 genes had a gene expression change nominally correlated with extent of improvement of disease activity variation measured with DAS28-CRP. Since the absolute correlation coefficients range between 0.53 and 0.73, these gene expression changes are not fully explained by modification of the disease activity. Gene enrichment analysis did not identify any significant pathways for these 19 genes. Nevertheless, one relevant biological process was found from data in the literature.

A decrease of disease activity at 6 months compared to baseline was correlated with an increase of expression level for TCAIM, TRAK1, KIA1279 (KIF1 binding protein), NDUFS1 and CCT5, which are involved in mitochondria processes [15–17]. *NDUFS1* encodes a mitochondrial protein NADH-ubiquinone oxidoreductase subunit, regulating reactive oxygen species (ROS) production [18]. These results are in accordance with the dysregulation of *CCT5* which encodes a molecular chaperone that is a member of the chaperonin containing TCP1 complex involved in autophagy, clearance of dysfunctional mitochondria and production of ROS [19]. Interestingly, in a previous study, we found a pre-silencing of the electron transport chain (ETC) pathway in R compared to NR before initiation of MTX/ABA [14]. The down regulation of genes expressed in mitochondria reflected the redox imbalance observed in R [14]. After 6 months of treatment, ABA significantly upregulated some ETC genes, including *NDUFS1*, in R probably leading to a slight increase in ROS, restoring the redox balance and improving T cell response as recently demonstrated by Yang Z [20]. This study has shown that MTX/ABA treatment modulates the gene expression involved in mitochondria processes and proliferation or apoptosis of cells. When we compared the gene expression of these 19 genes in healthy subjects and in patients with disease activity improvement, the gene expression of half of these genes dysregulated in RA patients fluctuated toward the normal gene expression level found in healthy subjects. The normalization of gene expression level under MTX/ABA treatment and the correlation between gene expression level and disease activity represent two arguments to consider the involvement of these genes in RA pathophysiology.

The gene expression level of the majority of genes with a significant variation of expression under MTX/ABA in R was not correlated with disease activity. This may be due to little variation (2.3–5.2) in the response amongst the R group. The submission of these genes to GO and Wikipathway databases found 20 significant biological processes and 6 signaling pathways.

ABA increased mRNA processes through two pathways, that seems relevant since Nakamura S *et al*. also found that dysregulation of genes was involved in RNA elongation arrest and recovery, regulation of apoptosis and formation of RNA polymerase II elongation complex [21].

Concerning TCR signaling pathway, the expression level of *GRB2* and *IKBKγ* decreased while *ATF2*, *CD247*, *LAT* and *CARD11* increased at 6 months compared to baseline under MTX/ABA. The growth factor receptor-bound protein 2 (GRB2) modulates and integrates signals from receptors on cellular surfaces in inner signaling pathways and is crucial for amplification of TCR signalling [22]. Moreover, IKBKγ, also called NEMO, exercises a negative action on TCR-induced NF-κB activation [22, 23]. On the contrary, *LAT*, *CD247*, *ATF2* and *CARD11* were overexpressed under MTX/ABA in R. Linker for activation of T cells (LAT) binds Grb2 to mediate T cell activation, proliferation and cytokine production [24]. The T-cell receptor T3 zeta chain (CD3ζ) or CD247 is essential for assembly, surface expression and signaling cascade of the T-cell receptor-CD3 (TCR/CD3) complex. A reduced density of synovial T-cell surface CD3ζ has been observed in RA patients, suggesting a decrease in TCR signaling, thus encouraging positive selection of autoreactive T effector cells in the thymus [25, 26]. While ABA is supposed to block T cell activation, our analysis of the TCR signaling pathway and the literature suggest a surprising activation of T cells. Normally, ABA binds CD80/86 to prevent the costimulatory signal relative to the interaction between CD28 and CD80/86, leading to T cell inactivation. Since T cells were activated in RA, the level of CTLA4 expressed on T cell membranes was increased. CTLA4 is also a ligand to CD80/86 to induce anti-proliferative effects on T cells [27]. Therefore, since ABA binds CD80/86, the interaction between CTLA4 and CD80/86 is blocked, inducing the suppressive effects of inhibition on T cell, leading finally to T cell activation. This presumed mechanism could explain the T cell activation observed in our study. Further functional analyses are necessary to confirm this new mechanism of action of ABA.

Protein degradation by the proteasome is one of the major regulatory mechanisms in the cell. In our study, 7 genes from this pathway (*UBC*, *PSMC1*, *PSMD6*, *HLA-B*, *HLA-C*, *HLA-G* and *HLA-J*) were mainly up-regulated in MTX/ABA responders at 6 months compared to baseline. These results are in agreement with the increase in mRNA processes. Indeed, proteasome activation may reflect the activation of mRNA processes since the ubiquitin-proteasome system has both proteolytic and non-proteolytic functions in multiple aspects of the transcription process, including initiation, elongation and mRNA processes [28].

*AIFM1*, *ATF2* and *CYCS* up-regulated in our study, are released from the mitochondrion intermembrane space into the cytosol and the nucleus and are pro-apoptotic factors. CYCS induces apoptosome formation involved in the cell death by caspase-dependent pathway, while AIFM1 contributes to apoptotic nuclear DNA damage in a caspase-independent way [29]. ATF2 and AIFM1 also induce mitochondria to release the apoptogenic proteins cytochrome c (CYCS). In addition, *CARD11* and *IKBKG* (also called NEMO) involved in TCR pathway, were dysregulated under ABA in favor of apoptosis since CARD11 is an upstream activator of BCL10 and NF-κB signaling. On the contrary, the gene expression of Fas-associated protein with Death Domain (*FADD*) was decreased under ABA enhancing cell survival. To explain the opposite effects of MTX/ABA in apoptosis, further studies are necessary in order to better understand the effect of ABA on different blood cells.

Finally, *GRB2*, *MMP9*, *TIMP2* were down-regulated under MTX/ABA at 6 months compared to baseline in RA responders, whereas *ATF2*, *PXN* and *VHL* were up-regulated. All these genes are involved in the angiogenesis process [30]. *GRB2* and *MMP9* are known to promote angiogenesis. Indeed, MMP9 is thought to play an important role in the degradation of extracellular matrix (ECM) and to facilitate the process which is useful for angiogenesis. The dysregulation of *VHL*, *GRB2* and *MMP9* suggests a decrease of angiogenesis at 6 months compared to baseline under MTX/ABA in R [31].

The limitation of this study was the low number of NR to MTX/ABA, the concomitant treatment of MTX making the interpretation of the own effects of ABA difficult. Moreover, the study was performed from the whole blood and not from cell subsets such as monocytes, T or B cells. RNA sequencing on each cell population would be more appropriated to understand the specific cellular effects of ABA.

## 5. Conclusions

The MTX/ABA concomitant treatment regulates few genes whose expression is correlated with extent of improvement of disease activity in RA responders. These genes are involved in inflammation and in mitochondrial processing suggesting the restoration of oxidant signaling. Most dysregulated genes at 6 months compared to baseline under MTX/ABA treatment were relative to drugs without disease activity correlation and were involved in RNA processes, TCR signaling, apoptosis, angiogenesis or proteasome pathways. Genes relative to TCR signaling were dysregulated in favor of T cell activation in responders to ABA. ABA, targeting CD80/86, blocks the T cell activation in RA which is usually mediated by the interaction of CD28 and CD80/86. ABA also blocks the T cell inhibition mediated by the dominant interaction of CTLA4 and CD80/86, resulting in T cell activation. Moreover, ABA might activate mRNA processing and proteasome with pro-apoptotic and anti-angiogenesis effects. Although further investigations are required to confirm these effects, this study already highlights new mechanisms of action of ABA associated with MTX in RA responders.

## Supporting information

S1 Fig Flow chart of rheumatoid arthritis patients selected from APPRAISE trial. (PDF)

S2 Fig Correlation analysis between variation of disease activity and changes of gene expression associated with MTX/ABA in responders. Pearson correlation coefficients (r) were calculated for each 672 transcripts wich are significantly dysregulated between baseline and 6 months. Fold change (FC; 6 months/baseline) of 19 transcripts were significantly (p<0.05) correlated to variation of disease activity (D DAS). A_24_P635355: Agilent probe ID; ARRB2: arrestin beta-2; CCS: copper chaperone for superoxide dismutase; CCT5: Chaperonin containing TCP1 subunit 5; EIF3F: eukaryotic translation initiation factor 3 subunit; F8: Coagulation factor VIII; ING3: inhibitor of growth family member 3; KIAA1279: KIF1 binding protein; LLPH: LLP homolog, long-term synaptic facilitation; NDUFS1: NADH-ubiquinone oxidoreductase 75 kDa subunit; NREP: neuronal regeneration related protein; PAXBP1: PAX3 and PAX7 binding protein 1; PWP1: Periodic tryptophan protein 1 omolog; SCAPER: S-phase cyclin A-associated protein in the endoplasmatic reticulum; TCAIM: T-cell activation inhibitor; TRAK1: Trafficking protein Kinesin binding 1; ZKSCAN7: Zinc finger with KRAB and SCAN domains 7; ZNF436: zinc finger protein 436.
(PDF)

S3 Fig Clinical and biological data from responders and no-responders rheumatoid arthritis patients. CRP: C reactive protein; DAS28: disease activity score 28; NR: no-responders; R: responders; VAS: visual analog scale.
(PDF)
