# Supplementary figures and images for: Gene expression regulated by abatacept associated with methotrexate and correlation with disease activity in rheumatoid arthritis
Source: PLoS One. 2020 Aug 6;15(8):e0237143. doi: 10.1371/journal.pone.0237143 (PMC7410313; doi:10.1371/journal.pone.0237143)

# Methotrexate/Abatacept treated RA patients

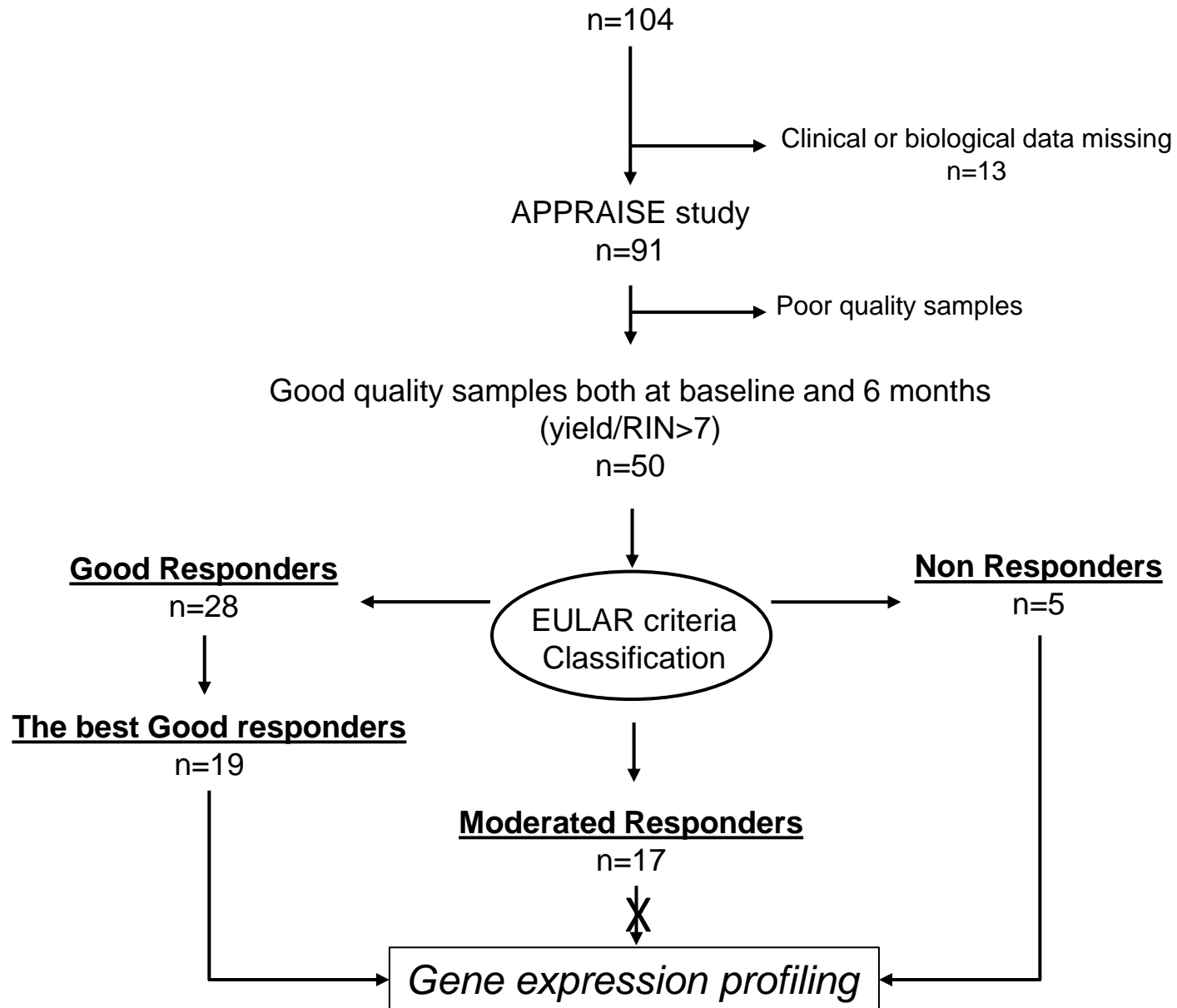

Supplement: S1 Fig — (PDF) [file pone.0237143.s001.pdf]
