# Supplementary material for: Gene expression regulated by abatacept associated with methotrexate and correlation with disease activity in rheumatoid arthritis
Source: PLoS One. 2020 Aug 6;15(8):e0237143. doi: 10.1371/journal.pone.0237143 (PMC7410313; doi:10.1371/journal.pone.0237143)
Supplement: S2 Fig — Pearson correlation coefficients (r) were calculated for each 672 transcripts wich are significantly dysregulated between baseline and 6 months. Fold change (FC; 6 months/baseline) of 19 transcripts were significantly (p<0.05) correlated to variation of disease activity (D DAS). A_24_P635355: Agilent probe ID; ARRB2: arrestin beta-2; CCS: copper chaperone for superoxide dismutase; CCT5: Chaperonin containing TCP1 subunit 5; EIF3F: eukaryotic translation initiation factor 3 subunit; F8: Coagulation factor VIII; ING3: inhibitor of growth family member 3; KIAA1279: KIF1 binding protein; LLPH: LLP homolog, long-term synaptic facilitation; NDUFS1: NADH-ubiquinone oxidoreductase 75 kDa subunit; NREP: neuronal regeneration related protein; PAXBP1: PAX3 and PAX7 binding protein 1; PWP1: Periodic tryptophan protein 1 omolog; SCAPER: S-phase cyclin A-associated protein in the endoplasmatic reticulum; TCAIM: T-cell activation inhibitor; TRAK1: Trafficking protein Kinesin binding 1; ZKSCAN7: Zinc finger with KRAB and SCAN domains 7; ZNF436: zinc finger protein 436. (PDF) [file pone.0237143.s002.pdf]

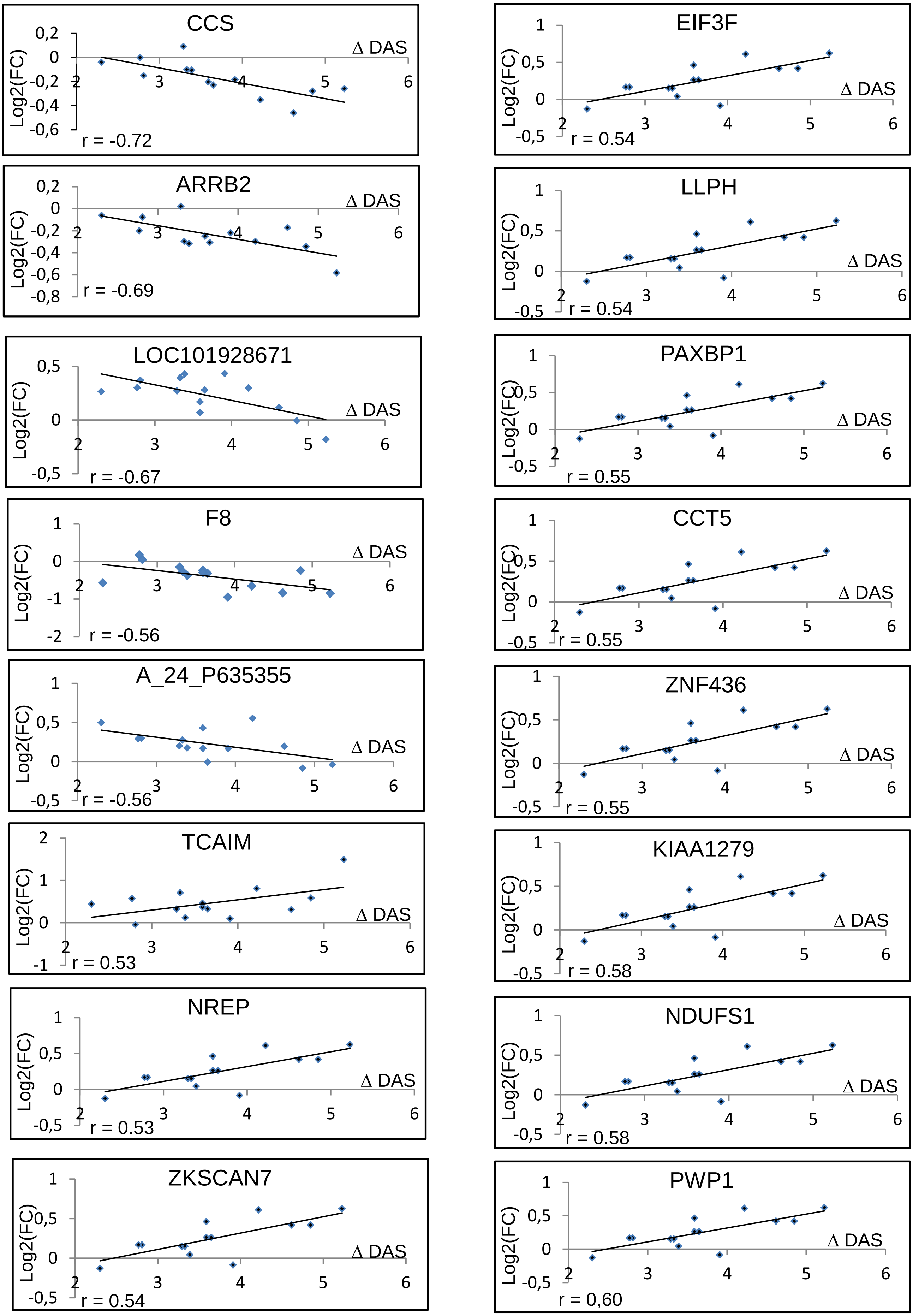
CCS
Log2(FC)
Δ DAS
r = -0.72
EIF3F
Log2(FC)
Δ DAS
r = 0.54
ARRB2
Log2(FC)
Δ DAS
r = -0.69
LLPH
Log2(FC)
Δ DAS
r = 0.54
LOC101928671
Log2(FC)
Δ DAS
r = -0.67
PAXBP1
Log2(FC)
Δ DAS
r = 0.55
F8
Log2(FC)
Δ DAS
r = -0.56
CCT5
Log2(FC)
Δ DAS
r = 0.55
A_24_P635355
Log2(FC)
Δ DAS
r = -0.56
ZNF436
Log2(FC)
Δ DAS
r = 0.55
TCAIM
Log2(FC)
Δ DAS
r = 0.53
KIAA1279
Log2(FC)
Δ DAS
r = 0.58
NREP
Log2(FC)
Δ DAS
r = 0.53
NDUFS1
Log2(FC)
Δ DAS
r = 0.58
ZKSCAN7
Log2(FC)
Δ DAS
r = 0.54
PWP1
Log2(FC)
Δ DAS
r = 0,60

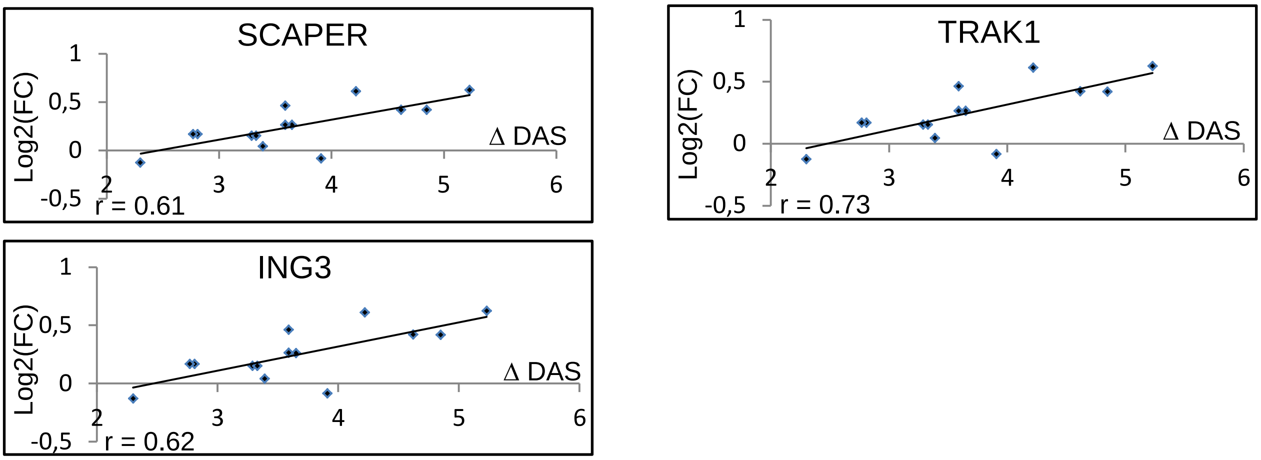

figure S1 : correlation analysis between variation of disease activity and fluctuation of gene expression associated with MTX/ABA in responders. Pearson correlation coefficients (r) were calculated for each 672 transcripts wich are significantly dysregulated between baseline and 6 months. Fold change (FC; 6 months/baseline) of 19 transcripts were significantly ($p<0.05$) correlated to variation of disease activity (Δ DAS).

A_24_P635355 : Agilent probe ID ; ARRB2 : arrestin beta-2 ; CCS : copper chaperone for superoxide dismutase ; CCT5 : Chaperonin containing TCP1 subunit 5 ; EIF3F : eukaryotic translation initiation factor 3 subunit ; F8 : Coagulation factor VIII ; ING3 : inhibitor of growth family member 3 ; KIAA1279 : KIF1 binding protein ; LLPH : LLP homolog, long-term synaptic facilitation ; NDUFS1 : NADH-ubiquinone oxidoreductase 75 kDa subunit ; NREP : neuronal regeneration related protein ; PAXBP1 : PAX3 and PAX7 binding protein 1 ; PWP1: Periodic tryptophan protein 1 homolog ; SCAPER : S-phase cyclin A-associated protein in the endoplasmatic reticulum ; TCAIM: T-cell activation inhibitor ; TRAK1: Trafficking protein Kinesin binding 1 ; ZKSCAN7 : Zinc finger with KRAB and SCAN domains 7 ; ZNF436: zinc finger protein 436.
