# Supplementary material for: Gene expression regulated by abatacept associated with methotrexate and correlation with disease activity in rheumatoid arthritis
Source: PLoS One. 2020 Aug 6;15(8):e0237143. doi: 10.1371/journal.pone.0237143 (PMC7410313; doi:10.1371/journal.pone.0237143)
Supplement: S3 Fig — CRP: C reactive protein; DAS28: disease activity score 28; NR: no-responders; R: responders; VAS: visual analog scale. (PDF) [file pone.0237143.s003.pdf]

Patient Id	Abatacept response	Age (year)	Sex (F/M)	Disease duration (year)	Methotrexate dose (mg/week)	Corticosteroid dose (mg/day)	Tender joint count/28	Swollen joint count/28	Patient assessment of disease (VAS scale/100mm)	DAS28-CRP	CRP (mg/L)
56	Responder	61	F	3	10	7,5	26	21	90	7,51	23,7
153	Responder	62	M	0	25	0	19	18	65	7,04	71,9
76	Responder	64	F	17	15	5	20	20	68	6,07	2,05
121	Responder	20	F	1	15	5	24	24	59	6,48	4,06
9	Responder	45	F	1	10	4	12	18	28	5,14	5,24
8	Responder	69	F	0	10	0	12	7	71	5,23	4,2
52	Responder	55	M	19	15	4	17	5	95	6,19	13,6
155	Responder	29	F	12	15	0	12	11	98	6,13	12,1
141	Responder	72	M	0	15	0	15	10	59	5,85	15,6
133	Responder	67	F	0	20	0	13	13	23	4,89	3,99
138	Responder	49	F	3	15	5	14	8	75	5,60	6,03
105	Responder	82	F	1	15	10	9	6	19	4,71	21,1
115	Responder	63	F	5	20	0	10	9	74	5,27	5,34
57	Responder	85	F	1	15	7,5	8	9	45	4,64	4,77
1	No-Responder	58	M	15	20	0	17	10	76	6,25	16,8
42	No-Responder	74	M	3	20	8	3	8	63	4,93	38,3
72	No-Responder	59	F	1	15	5	23	16	62	6,99	42,1
3	No-Responder	51	F	3	15	0	3	7	6	2,96	0,77
93	No-Responder	51	F	22	20	0	2	6	40	3,60	4,39
